# Performance of ChatGPT-4 and Bard chatbots in responding to common patient questions on prostate cancer ^177^Lu-PSMA-617 therapy

**DOI:** 10.3389/fonc.2024.1386718

**Published:** 2024-07-12

**Authors:** Gokce Belge Bilgin, Cem Bilgin, Daniel S. Childs, Jacob J. Orme, Brian J. Burkett, Ann T. Packard, Derek R. Johnson, Matthew P. Thorpe, Irbaz Bin Riaz, Thorvardur R. Halfdanarson, Geoffrey B. Johnson, Oliver Sartor, Ayse Tuba Kendi

**Affiliations:** ^1^ Department of Radiology, Mayo Clinic, Rochester, MN, United States; ^2^ Division of Medical Oncology, Department of Oncology, Mayo Clinic, Rochester, MN, United States; ^3^ Department of Hematology and Oncology, Mayo Clinic, Phoenix, AZ, United States; ^4^ Department of Immunology, Mayo Clinic, Rochester, MN, United States; ^5^ Department of Urology, Mayo Clinic, Rochester, MN, United States

**Keywords:** prostate cancer, 177 Lu-PSMA-617 therapy, ChatGPT, Bard, artificial intelligence, machine learning, chatbot, information literacy

## Abstract

**Background:**

Many patients use artificial intelligence (AI) chatbots as a rapid source of health information. This raises important questions about the reliability and effectiveness of AI chatbots in delivering accurate and understandable information.

**Purpose:**

To evaluate and compare the accuracy, conciseness, and readability of responses from OpenAI ChatGPT-4 and Google Bard to patient inquiries concerning the novel ^177^Lu-PSMA-617 therapy for prostate cancer.

**Materials and methods:**

Two experts listed the 12 most commonly asked questions by patients on ^177^Lu-PSMA-617 therapy. These twelve questions were prompted to OpenAI ChatGPT-4 and Google Bard. AI-generated responses were distributed using an online survey platform (Qualtrics) and blindly rated by eight experts. The performances of the AI chatbots were evaluated and compared across three domains: accuracy, conciseness, and readability. Additionally, potential safety concerns associated with AI-generated answers were also examined. The Mann-Whitney U and chi-square tests were utilized to compare the performances of AI chatbots.

**Results:**

Eight experts participated in the survey, evaluating 12 AI-generated responses across the three domains of accuracy, conciseness, and readability, resulting in 96 assessments (12 responses x 8 experts) for each domain per chatbot. ChatGPT-4 provided more accurate answers than Bard (2.95 ± 0.671 vs 2.73 ± 0.732, *p*=0.027). Bard’s responses had better readability than ChatGPT-4 (2.79 ± 0.408 vs 2.94 ± 0.243, *p*=0.003). Both ChatGPT-4 and Bard achieved comparable conciseness scores (3.14 ± 0.659 vs 3.11 ± 0.679, *p*=0.798). Experts categorized the AI-generated responses as incorrect or partially correct at a rate of 16.6% for ChatGPT-4 and 29.1% for Bard. Bard’s answers contained significantly more misleading information than those of ChatGPT-4 (*p* = 0.039).

**Conclusion:**

AI chatbots have gained significant attention, and their performance is continuously improving. Nonetheless, these technologies still need further improvements to be considered reliable and credible sources for patients seeking medical information on ^177^Lu-PSMA-617 therapy.

## Introduction

Following pivotal clinical trial, ^177^-LuPSMA-617 (Pluvicto) molecular targeted radioligand therapy has gained significant momentum and is rapidly becoming cornerstones in patient management (1). As ^177^-LuPSMA-617 therapy has been shown to prolong overall survival in addition to progression free survival, have mostly mild side effects, and improve quality of life, an increasing number of patients are opting for this treatment. Before finalizing their decision, patients seek to learn more about the theragnostic approach and its potential benefits in their individual cases. Therefore, it is expected that a growing number of patients may turn to artificial intelligence (AI) chatbots for information and guidance regarding these novel treatment options (2, 3).

Recent advances in AI-driven systems and their ability to generate sophisticated responses for almost any prompt have revolutionized communication and access to information (4). With ongoing innovations over the past decades, AI chatbots are now capable of interacting in a human-like manner. This evolution has fostered trust among individuals who perceive AI-driven systems as credible. As a result, AI-driven systems are now increasingly utilized for decision-making across various facets of daily life.

Particularly after the emergence of advanced large language models such as OpenAI ChatGPT (Chat-Generative Pre-Trained Transformer), and Google Bard, AI chatbots have attracted significant attention and are being widely adopted. These AI chatbots could potentially serve as a valuable resource for patients, who are seeking answers to treatment options, even before consulting with healthcare providers. It is also important to note that all AI chatbots are still under development and may produce fabricated answers, which are commonly referred to as hallucinations (5–7). Moreover, there is currently no established tool to detect fabricated or misleading information in AI chatbot responses (8). Therefore, the merits and limitations of new AI chatbots in providing medical information has been a topic of substantial interest.

In this study, we aim to assess the performance of popular AI chatbots, ChatGPT-4 and Bard, in addressing patient inquiries about ^177^-LuPSMA-617 therapy to understand the user experience and to identify areas for improvement.

## Materials and method

### Data collection

Since no patient data were used, this study was exempt from the requirements of the IRB.

Two experts, consisting of a nuclear radiologist (ATK) and an oncologist-nuclear medicine specialist (OS), listed the twelve most commonly asked questions by patients on ^177^-LuPSMA-617 therapy (**Table 1**). Subsequently, these questions were prompted to an author-owned OpenAI ChatGPT-4 and Google Bard on October 8, 2023. All questions were asked to chatbots by one investigator to provide consistency. Since AI chatbots can learn as they interact with users and their questions/instructions, each question was asked in a separate chat box to eliminate potential memory retention bias. Subsequently, a two-part questionnaire designed by using an online survey platform, Qualtrics (Provo, UT), to evaluate chatbot performance (9). Each part contained chatbots’ answers to the same set of 12 questions.

**Table 1:** Potential patient’s questions regarding LuPSMA.

All 12 Questions Prompted to ChatGPT and Bard	
**Q1.**	How does Pluvicto/LuPSMA therapy work?
**Q2.**	How are patients selected to be treated with Pluvicto/LuPSMA therapy?
**Q3.**	Who is most likely to benefit from Pluvicto/LuPSMA therapy?
**Q4.**	How can I prepare for Pluvicto/LuPSMA therapy?
**Q5.**	How Pluvicto/LuPSMA is administered?
**Q6.**	What are the most common side effects of Pluvicto/LuPSMA therapy?
**Q7.**	What instruction should I receive from physicians before and after Pluvicto/LuPSMA therapy?
**Q8.**	How many doses of Pluvicto/LuPSMA should I receive?
**Q9.**	How do physicians monitor the effectiveness of Pluvicto/LuPSMA therapy?
**Q10.**	How soon will I know if the treatment is effective?
**Q11.**	Where can I get Pluvicto/LuPSMA therapy?
**Q12.**	How much does Pluvicto/LuPSMA therapy cost?

Q, Question.

**Figure 1:**
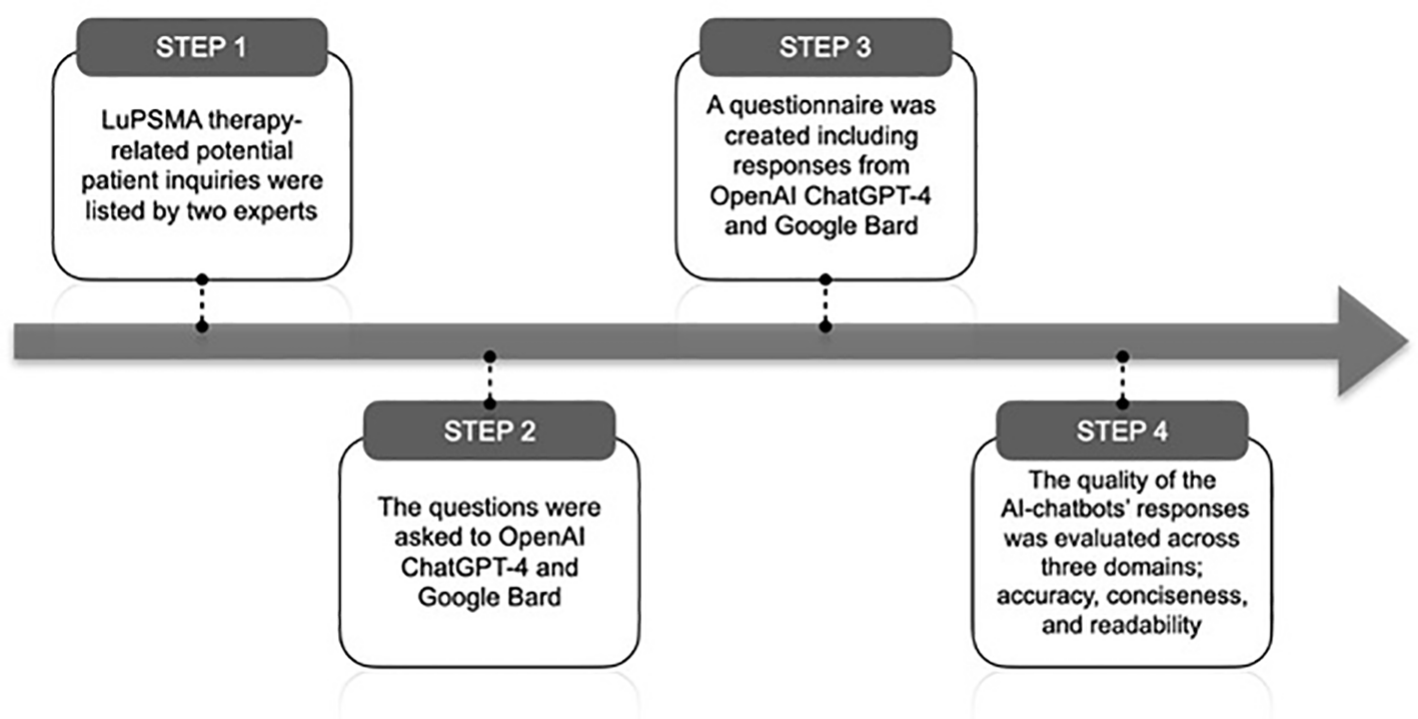
Methodology of the study.

The questionnaire was then circulated among eight experts and the assessment was conducted in a blinded manner. The quality of the chatbots’ responses was evaluated across three domains: accuracy, conciseness, and readability (**Figure 1**). For accuracy and conciseness, a 4-point scale was utilized, while readability was assessed on a 3-point scale. Both AI chatbots grades on 3 domains were compared using statistical analyses. Additionally, responses with an accuracy score of ≤ 2 were categorized as incorrect/misleading answers, and potential safety concerns associated with AI-generated answers were examined.

Four nuclear radiologists and four oncologists independently evaluated the responses provided by ChatGPT-4 and Bard. The rating scale used for assessment is detailed in **Table 2**.

**Table 2:** Parameters used for survey.

Parameters	Description
Accuracy	
**1**	Completely incorrect or misleading answer
**2**	Partially correct answer, but may contain some errors or omissions
**3**	Mostly correct answer, but may be missing some details or could be improved some way
**4**	Completely correct and comprehensive
Conciseness	
**1**	Very wordy and lengthy
**2**	Generally wordy and lengthy, could be more concise
**3**	Potentially concise and as long as it needs to be
**4**	Very concise as short as possible without sacrificing completeness and accuracy
Readability	
**1**	Very difficult to read and understand. Full of medical jargon and complex sentences
**2**	Partially difficult to read and understand. Some medical jargon and complex sentences
**3**	Easy to read and understand. Written in simple language and avoids medical jargon

### Statistical analysis

Statistical analyses were conducted with SPSS (version 25.0 for Windows; Illinois, USA). The Mann-Whitney U test was used to for nonparametric data to compare the scores of the ChatGPT-4 and Bard in three domains. The Chi-square test was utilized for categorical data to compare the rates of medically incorrect information in AI chatbots’ answers. Descriptive statistics were also calculated for each variable. A *p*-value threshold of.05 was used to determine statistical significance in this study.

## Results

Eight experts participated in the survey, evaluating 12 AI-generated responses across the three domains of accuracy, conciseness, and readability, resulting in 96 assessments (12 responses x 8 experts) for each domain per chatbot.

The Mann Whitney U test was used to compare the scores of the ChatGPT-4 and Bard. ChatGPT answers were scored to be more accurate (accuracy scores = 2.95 ± 0.671 vs 2.73 ± 0.732, ChatGPT vs Bard respectively, *p*=0.03). Bard’s responses were evaluated to have better readability than ChatGPT-4 (readability scores = 2.79 ± 0.408 vs 2.94 ± 0.243, ChatGPT vs Bard respectively, *p*=0.003). Both ChatGPT-4 and Bard were ranked with comparable conciseness scores (3.14 ± 0.659 vs 3.11 ± 0.679, respectively, *p*=0.80).

**Figure 2:**
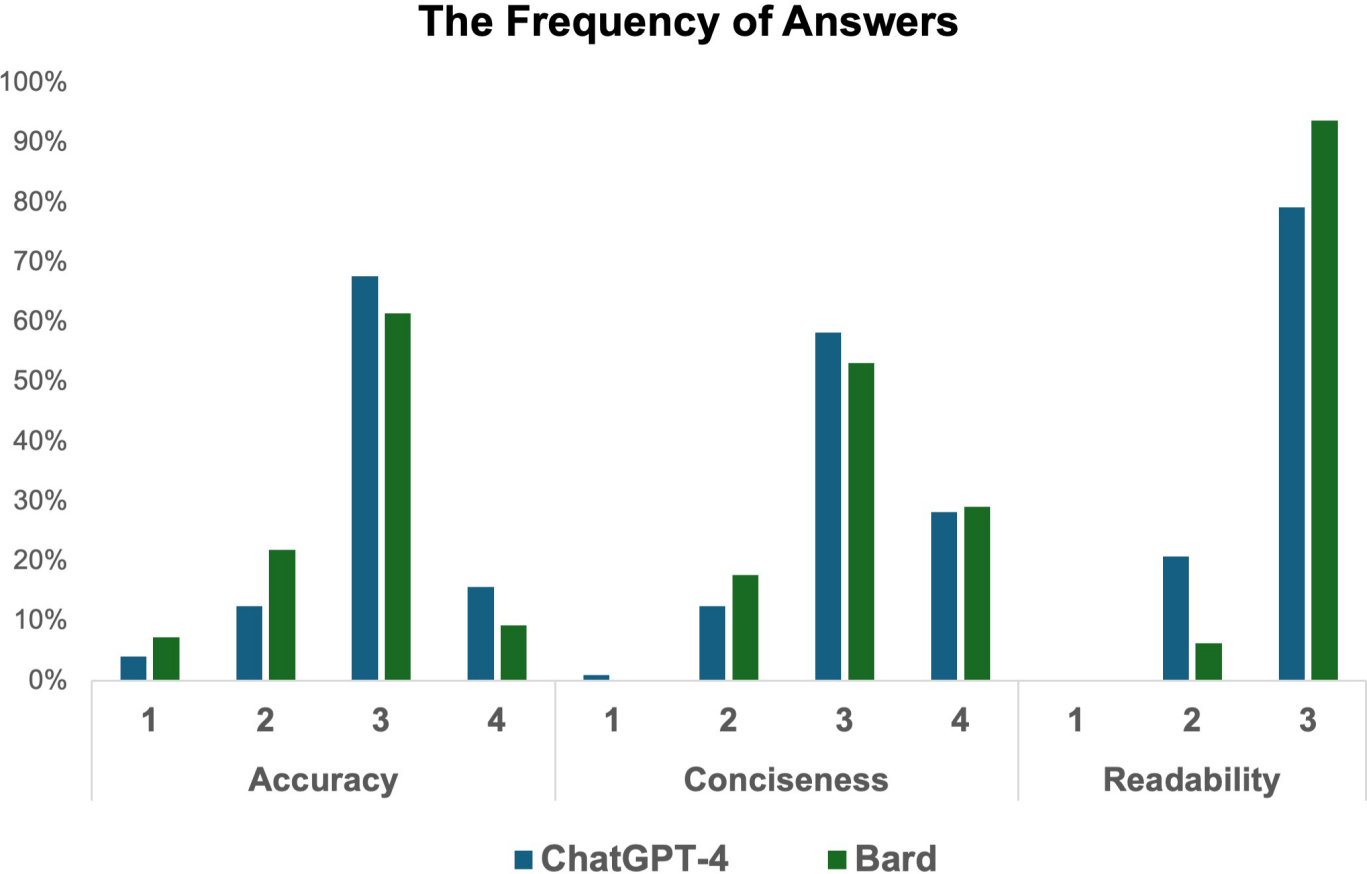
Frequency of answers.

The Chi-square test was utilized to compare the rates of medically incorrect information in AI chatbots’ answers. Experts categorized the AI-generated responses as incorrect or partially correct at a rate of 16.6% (16/96) for ChatGPT-4 and 29.1% (28/96) for Bard. Bard’s answers contained significantly more incorrect or misleading information than those of ChatGPT-4 (29.1% vs 16.6%, respectively, p = 0.04). Detailed results are presented in **Tables 3** and **Figure 2**.

**Table 3:** Statistical results of AI-generated responses.

	Accuracy		Conciseness		Readability	
	ChatGPT-4	Bard	ChatGPT-4	Bard	ChatGPT-4	Bard
**Median**	3	3	3	3	3	3
**Mean**	2.95	2.73	3.14	3.11	2.79	2.94
**SD**	0.671	0.732	0.659	0.679	0.408	0.243
**Mean Rank**	104.06	88.94	97.42	95.58	89.50	103.50
**Sum of Rank**	9990.0	8538.0	9352.0	9176.0	8592.0	9936.0
**Mann Whitney U**	3882.0		4520.0		3936.0	
***p* value**	0.27		0.798		0.003	

SD, Standard Deviation.

## Discussion

Our study identified several important findings. First, ChatGPT-4 provided significantly more accurate answers than Bard, as judged by subject matter experts. Second, Bard’s responses had significantly better readability than those of ChatGPT-4. Third, both chatbots achieved comparable scores in terms of conciseness. Fourth, experts categorized the AI-generated responses as incorrect or partially correct at a rate of 17% for ChatGPT-4 and at a rate of 29% for Bard. These findings are important as they suggest AI chatbots have considerable potential to address common patient inquiries about theragnostic. However, our results also indicate that the current versions of AI chatbots may present misinformation, potentially posing a safety risk for patients seeking medical information, which are concordant with other literature on the topic (10–12).

Safety concerns associated with patients’ use of AI chatbots have become a topic of growing interest. Goodman et al. examined the accuracy of GPT-3.5 and GPT-4 responses to questions from various medical disciplines through a survey (10). Their results suggested that ChatGPT has potential for delivering accurate and detailed medical information while it may prone to generate hallucinating responses that include partially or completely incorrect. Additionally, their study demonstrated that almost half of the responses still had inaccuracies or omissions that could potentially pose safety concerns. In the existing literature, the majority of studies have focused solely on ChatGPT, while other AI chat tools like Bard and Bing have received less attention. A study by Janopaul-Naylor, et al. assessed response quality of ChatGPT 3.5 and Bing to typical patient questions about a variety of cancers (11). In their study, ChatGPT 3.5 performed better on questions related to breast, lung, colorectal, and prostate cancer. Nevertheless, both ChatGPT and Bing occasionally provided completely inaccurate or contradictory answers. Similarly, another study by Rahsepar et al. evaluated the performance of ChatGPT-3.5, Bard, Bing, and Google search engines in answering patient questions about lung cancer prevention and screening (12). Although ChatGPT-3.5 generally demonstrated superior accuracy, neither chatbot could generate completely accurate responses. In the same study, the authors also prompted chatbots to obtain the explanation of various radiological terminologies that could appear in a patient’s imaging test results such as Lung RADS (Lung Imaging Reporting and Data System) classification. When they posed questions regarding hypothetical Lung RADS categories (Lung RADS 5 or Lung RADS 6), Bard produced fabricated answers and provided survival rates for these nonexistent categories. Such misleading and inaccurate information could adversely affect patients’ decision-making processes and compromise optimal patient care. Therefore, screening radionuclide therapy patients regarding the use of AI chatbots for medical information may be valuable to ensure accurate understanding of treatment plans and post-treatment care.

Although neither tool produces accurate and reliable responses, in current literature the performance of Bing or Bard is falling behind ChatGPT in terms of accuracy and reliability in the medical field, which may be attributable to their distinct training models. All three platforms, ChatGPT, Bard, and Bing, are pre-trained on extensive text and code datasets, but they differ in methodology. ChatGPT utilizes ‘Reinforcement Learning from Human Feedback’, involving interaction with human trainers who refine its responses. This method, along with additional fine-tuning by human-generated text and code, enhances ChatGPT’s accuracy and conversational capability. In contrast, Bard and Bing use ‘Transformer-Based Masked Language Modeling’ in which the model was trained to predict the missing words in a sequence of words by prioritizing satisfaction of users. Also, Bard and Bing have real-time internet access which allows them to generate the most up-to-date information. However, as the training methods of Bard and Bing do not involve fine-tuning or getting help from human trainers, they might provide unfiltered and more uncontrolled information and it might cause inaccuracy of responses. On the other hand, both ChatGPT 3.5 and ChatGPT 4 are limited by the nature of their training data, which only extends up to September 2021 and January 2022, respectively. Thus, they are unable to generate responses beyond the stated dates.

The American Medical Association recommends simplifying patient-directed information to a sixth-grade level of English comprehension to facilitate communication (13). Maintaining high readability through the use of simple, straightforward language is crucial for AI chatbots to ensure that the information provided is clearly understood by patients, avoiding any confusion or misinterpretation. In our study, although the conciseness scores were comparable, Bard performed better at providing easily readable responses without compromising the integrity of the information. Haver et al. conducted a study comparing both versions of ChatGPT and Bard for the readability of answers to frequently asked questions about lung cancer and screening (14). They graded readability using the Flesch Reading Ease scale and determined the U.S. education grade level readability with an online tool. Similarly, in their study, Bard responses had better readability as compared to ChatGPT. However, overall average readability was still too challenging for the average adult patient. Similarly, Musheyev et al. evaluated the quality of information provided by four AI chatbots including ChatGPT, Perplexity, Chat Sonic, and Microsoft Bing AI, regarding questions related to prostate, bladder, kidney, and testicular cancers (15). Their findings were also in line with our result and the literature. In their study, the AI chatbots generally provided mostly accurate and moderate-high quality of responses. Nonetheless, the clarity of the responses was lacking, and the reading levels often were higher than the recommended threshold for patient-facing health information.

As AI technology and its applications, including chatbots, become more common in daily life, new ethical concerns are emerging related to the use of AI chatbots for generating and spreading information. For instance, individuals may entrust AI chatbots with sensitive medical data; thus, ensuring data anonymization and adherence to privacy regulations is of paramount importance (16). Additionally, navigating the intricacies of accountability and liability in AI-generated responses poses challenges, necessitating clear guidelines and regulations to allocate responsibility between developers and users. Another concern might be that AI responses can be biased and one-sided depending on the training dataset (17, 18). Therefore, ensuring fairness, comprehensiveness, and equity in the training dataset is also essential to avoid potential biases. These concerns are examples of potential ethical implications that necessitate collaborative efforts among stakeholders to identify these issues, create solutions, and establish legal regulations.

Our study has several limitations. Firstly, the dataset comprised 12 questions prepared by 2 physicians, which may not fully represent the diversity of patient inquiries and might introduce bias. Secondly, our survey was limited to responses from 8 physicians, which might not fully capture the perspectives of the broader medical community. Thirdly, although we asked the same 12 questions to both chatbots, it is important to note that the quality of AI-generated responses might be influenced by the phrasing used in the prompts. Lastly, AI chatbots were evaluated at a single time point; since they are regularly updated, they can perform better in future assessments.

Overall, AI-chatbots have drawn great attention and their performances improve every day. However, these tools still require further refinement to become trusted assets for patients to access reliable medical information. Therefore, future collaborative studies involving healthcare professionals and AI developers are essential to fully harness the potential of AI technologies in healthcare. These studies could focus on optimizing AI chatbots for medical support, ensuring they present accurate and reliable answers tailored to the specific needs of patients.

## Data availability statement

The datasets presented in this study can be found in online repositories. The names of the repository/repositories and accession number(s) can be found below: https://openai.com/blog/chatgpt.

## Author contributions

GB: Methodology, Software, Writing – original draft, Writing – review & editing. CB: Methodology, Writing – original draft, Writing – review & editing. DC: Writing – review & editing. JO: Writing – review & editing. BB: Writing – review & editing. AP: Writing – review & editing. DJ: Writing – review & editing. MT: Writing – review & editing. IR: Writing – review & editing. TH: Writing – review & editing. GJ: Writing – review & editing. OS: Supervision, Writing – review & editing. AK: Supervision, Writing – review & editing.
